# Infection and re-infection of *Leptospira* spp. in stray dogs and cats from Bogota, Colombia

**DOI:** 10.14202/vetworld.2024.973-980

**Published:** 2024-05-04

**Authors:** María Margarita Molina Puentes, Karen Daniela Jaimes Camargo, Yuly Angélica Monroy Roberto, Blanca Lisseth Guzman-Barragan, Gabriel Andrés Tafur-Gomez, Nelson Fernando Santana Clavijo

**Affiliations:** Department of Veterinary Medicine, Universidad de Ciencias Ambientales y Aplicadas (UDCA), Bogota, 111166, Colombia

**Keywords:** cat, dog, *Leptospira* spp, microagglutination test, phylogenetic analysis, polymerase chain reaction

## Abstract

**Background and Aim:**

Leptospirosis is a re-emerging zoonosis that is under-reported in tropical countries, and canines can be a potential reservoir of the disease. The objective of this study was to diagnose *Leptospira* spp. that is actively infected and re-infected in stray dogs and cats from Bogota, D.C., Colombia.

**Materials and Methods:**

A sample of 200 animals, including dogs and cats from the animal protection programs of Bogota, Colombia, were used in this study. Blood was collected from these animals for serum and DNA analysis. Conventional polymerase chain reaction (PCR) was performed using the 16s rRNA primer set, and higher-quality amplification products were sequenced by Sanger. For serodiagnosis, a group of PCR-positive samples was tested using the microagglutination test (MAT).

**Results:**

The overall PCR positivity of stray dogs and cats was 56%, 52.9%, and 65.3% in dogs and cats, respectively. The MAT seropositivity was 77.3%, and only dogs showed titers higher than 1:400. *Canicola*, *Icterohaemorrhagiae*, *Pomona*, *Hardjo Prajitno*, and *Canicola* and *Hardjo prajitno* were the serogroups associated with dogs and cats, respectively. Phylogenetic analysis revealed that the strains belonging to *Leptospira interrogans* serovars related to isolated samples of American, European, and Asian bats (*Myotis myotis*), dogs, and bovines of American origin.

**Conclusion:**

These results showed that stray dogs and cats were previously exposed to different serovars of *Leptospira* spp. and re-infected with other serovars that actively participated in the transmission cycle. These findings highlight the importance of actively diagnosing infectious animals to design effective intervention strategies.

## Introduction

Leptospirosis is a zoonotic disease caused by bacteria belonging to the genus *Leptospira* spp. The genus *Leptospira* has more than 20 species and 250 serovars that infect several species of mammals worldwide [1]. Leptospirosis is a re-emerging disease with complex epidemiologic dynamics and high underreporting, making it difficult to control [2]. Infection can be caused by humans’ direct or indirect exposure to leptospire-contaminated environments or animal reservoirs [1, 2]. The disease is related to different risk factors, such as rodents, water, occupational activities, animal production, poor sanitary conditions, and climate [3]. It shows a cosmopolitan distribution with more impact in tropical and developing countries. It is estimated that 1.03 million people are infected with leptospirosis worldwide each year, causing 58,900 deaths [4].

Domestic animals, such as pets, are one of the most important reservoirs of infection in urban environments due to their close contact with humans. The disease in dogs may be asymptomatic or present with acute anicteric illness and an icteric form [5]. Dogs are maintenance hosts for *Canicola* serovar; however, other serogroups such as *Bratislava*, *Grippotyphosa*, and *Pomona* cause incidental infection [5]. Although there are few studies on the involvement of cats in the epidemiology of leptospirosis, these animals develop a productive infection with kidney lesions or liver disease that sheds bacteria through urine and feces [6]. A previous study by Dorsch *et al*. [7] reported the culture growth of leptospires from the feline urine of naturally infected cats in South America, showing that this excretion of pathogenic *Leptospira* spp. is an underestimated source of infection.

In recent years, leptospirosis has been included in the list of mandatory reporting diseases in Colombia due to the high number of underreported cases, and some studies suggest an interface between animals and the environment [8]. In this transmission cycle, public health authorities should consider the increasing number of dogs on the streets, which is growing worldwide, especially in developing countries and in conflict zones, becoming an important reservoir for leptospirosis [9]. Similarly, pets were abandoned during the COVID-19 pandemic, which increased the number of animals in the streets, resulting in the participation of bacteria in the transmission [10].

In the diagnosis of pets, previous surveys in Colombia have used a microagglutination test (MAT) to detect previously infected animals whose antibody titers have been detected according to the serovar panel used in the assay. However, it is difficult to distinguish infectious antibodies produced during a recent or current infection from post-vaccine antibodies using this test. Polymerase chain reaction (PCR) tests can be used to diagnose actively infected pets with the highest bacteremia loads, identify the serovars involved, and could be used as a definitive test [11]. To identify actively infected and re-infected animals, both tests can be used to identify dogs and cats that maintain the transmission cycle. This study aimed to diagnose actively infected and re-infected *Leptospira* spp. in stray dogs and cats from Bogota, Colombia, with close human contact.

## Materials and Methods

### Ethical approval and Informed consent

The animals used in this study were handled and treated under qualified veterinary supervision in accordance with the animal experimentation rules described in the International Guiding Principles for Veterinary Research Involving Animals. Written informed consent was obtained from the owners of the animals before their inclusion, and personal or farm information was not threatening according to Colombian habeas data laws. This study was approved by the Animal Research Bioethics Committee of the Universidad de Ciencias Aplicadas y Ambientales, UDCA, with act number 001/2021. The positive animals were treated, and the owners were informed of appropriate measures.

### Study period and location

The study was conducted from October 2021 and February 2022 in the city of Bogota, D.C., located in central Colombia, in the eastern part of the Andes Mountain Range, coordinates 4°35′56′′N 74°04′51′′W, at an average altitude of 2640 and 3548 masl [12]. Bogota DC has a total area of 1775 km^2^, divided into 20 municipalities and 1922 neighborhoods. According to the rabies public health program, the population of dogs and cats is estimated to be 1,084,214 and 66,467 individuals, respectively [13].

### Animals studied

A total of 200 animals were selected, considering a 90% confidence interval, 5% error, and an estimated population of 66,467 roaming dogs and cats under vulnerability status [14]. Animals entered the Instituto Distrital de Protección y Bienestar Animal of Bogota, animal adoption programs, animal abandonment, animal welfare, veterinary emergencies, animal abuse, and sterilization programs of various locations in Bogota. The inclusion criteria for animals included dog and cat species of different breeds and ages. The exclusion criteria were any animal with a history of vaccination against *Leptospira* spp.

### Sampling

Whole blood samples were collected from these animals in red-cap tubes. All samples were identified and transported to the molecular diagnostic laboratory of the Universidad de Ciencias Aplicadas y Ambientales (UDCA) under refrigeration at 4°C–8°C. To obtain plasma samples and red blood cells, the samples received in the laboratory were centrifuged at 1800× *g* for 5 min. Samples were stored in vials and frozen at –20°C for later processing.

### PCR

The diagnosis of active infection was made through a PCR test, which identifies the bacteria or nucleic acid in the blood, particularly during the first 10 days when the bacteria are present in high concentrations [15]. DNA was extracted using a Qiagen DNA blood and tissue kit® (QIAGEN, Germany). The 16S rRNA primer set (A, 5’-GGCGGCGCGTCTITAAACATG-3’; B, 5’-TTCCCCCCATTGAGCAAGATT-3’) was used to perform the PCR technique [16]. These sequences refer to the 16S rRNA gene of pathogenic *Leptospira* spp., of which oligonucleotides A and B correspond to nucleotides 38–57 and 328–369. Similarly, oligonucleotides C and D correspond to nucleotides 58–77 and 328–324, respectively, with an amplification of 281–331 bp. Denaturation at 95°C for 3 min followed by 40 cycles of denaturation at 95°C for 30 s, annealing at 60°C for 30 s, extension at 72°C for 30 s, and the final extension step at 72°C for 7 min. Regarding the endogenous control, PCR was performed using GAPDH F (5’-GCCGTGGAATTTGCCGT-3’) and GAPDH R (3’-GCCATCAATGACCCCTTCAT-5’) [17], which produces a 164-bp amplicon.

### MAT

MAT diagnosis was performed to identify the presence of antibodies from previous infections and different serovars, and convalescent titers for acute infectious disease diagnosis were performed 2–4 weeks after the acute titer [15]. A group of serum samples positive by convenience PCR were processed by MAT using a panel of *Leptospira* spp.; *Pomona*, *Canicola*, *Icterohaemorrhagiae*, and *Hardjo prajitno* serogroups. Titers >1:100 were considered seropositive, indicating previous infection, whereas titers >1:400 were interpreted as an acutely infected animal with clinical signs [18].

### Sequencing analysis

To provide substantial support to the results, 10 positive samples (6 dog samples and 4 cat samples) were randomly chosen to perform sequencing; they were amplified again into a final volume of 50 μL of 16S PCR, and the amplification was verified with 2 μL of the product in 1.5% agarose gel and then sent to the Sanger purification and sequencing service of the SSiGMol laboratory at the Universidad Nacional de Colombia. The sequences obtained were analyzed, and the positions with a Phred score >20 were considered to assemble the consensus sequence using BioEdit 7.2 Software (https://bioedit.software.informer.com/7.2/) [19]. Subsequently, these sequences were aligned using the Basic Local Alignment Search Tool nt tool in GenBank, compared with GenBank® sequence homologs, and downloaded and aligned using the Clustal W tool in BioEdit Sequence Alignment Editor 7.2.5 [19]. MEGA X Software (https://www.megasoftware.net/) [20] was used to determine the evolutionary model and build a maximum likelihood (ML) tree with 1000 Bootstrap replicates.

## Results

**Figure-1:**
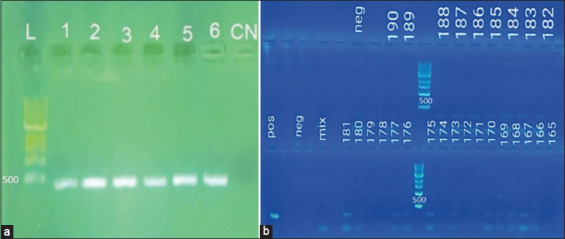
(a) DNA amplification of the positive controls from the extraction with the Qiagen DNA blood and tissue kit using the 16S primer set using the 1 kb marker. L bookmark 1 kb. (1) *Leptospira bratislava*. (2) *Leptospira canicola*. (3) *Leptospira gryppotyphosa*. (4) *Leptospira hardjo*. (5) *Leptospira icterohaemorrhagiae*. (6) *Leptospira pomona*. NC=Negative control. (b) polymerase chain reaction results of samples 165–190 from the extraction with the Qiagen DNA blood and tissue kit using the 16S primer set and using a 1 kb marker ladder size begin 500 kb.

**Figure-2:**
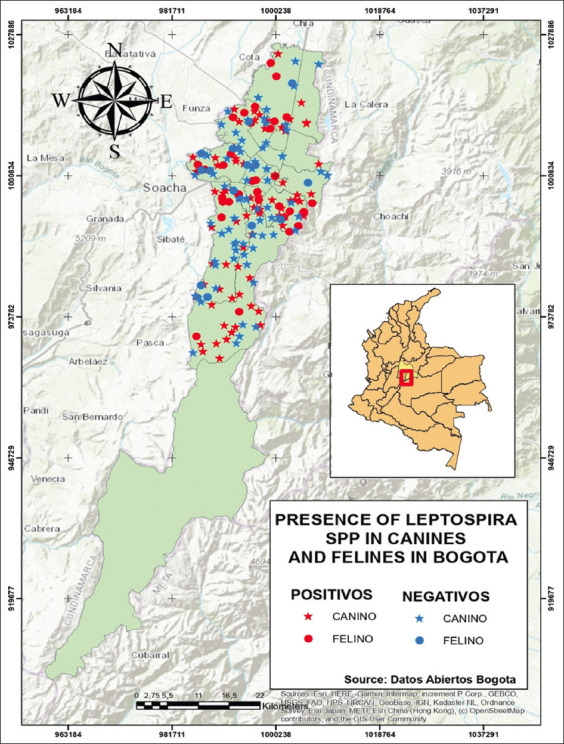
The location of cases with the presence of *Leptospira* spp. in stray dogs and cats in Bogota DC [Source: The map was generated using ArcGIS software].

Using the 16S primer set, the PCR results from 200 cat and dog samples revealed positivity for 112 (56%) of them (Figure-1). *Leptospira* spp. was found in 52.9% of dogs and 65.3% of cats, indicating a greater prevalence in felines. Female cats showed higher positivity rates than male cats, at 66.7%. Female dogs showed a higher positivity rate of 55.4% (Table-1). More cases have been reported in dogs aged between 9 and 12 years, accounting for 61.3% of all cases. However, there was a higher number of positives (67%) in early-aged cats aged between 0 and 4 years. More half-blood animals were analyzed than breed animals. However, in felines, half-blood animals presented a higher number of positive animals (54.4%) than breed animals (40%), and in canines, the percentage between the two groups was similar. Figure-2 shows the locations of cases of *Leptospira* spp. presence in stray dogs and cats throughout the city.

**Table 1:** Presence of *Leptospira* spp. in dogs and cats according to sex, age, and breed.

Variables	Total of cat	Positive animals	%	Total of dog	Positive animals	%
Sex						
Females	15	10	66.7	83	46	55.4
Males	34	22	64.7	68	34	50.0
Age						
0–4 years	31	21	67.7	58	32	55.2
5–8 years	13	8	61.5	40	21	52.5
9–12 years	5	3	60.0	31	19	61.3
13–16 years	0	0	0.0	22	8	36.4
Race						
Half blood	46	30	65.2	136	74	54.4
Breed animals	3	2	66.7	15	6	40.0

Half of the PCR-positive samples (66 samples) were processed using MAT (51 samples from dogs and 15 from cats). Of these, 51 (77%) tested positive (42 from dogs and 9 from cats), indicating previous exposure. However, 15 (23%) samples (9 from dogs and 6 from cats) were negative without previous exposure. Only 12% (8) of dogs had titers higher than 1:400, indicating the development of symptomatic disease, with 7 samples of *Canicola* and 1 sample of *Icterohaemorrhagiae*. Cats did not have titers >1:400. Dog samples tested positive for *Canicola* (36), *Hardjo Prajitno* (12), and four serogroups by MAT, *Icterohaemorrhagiae* (11), and *Pomona* (2). In contrast, cat samples showed positive results only for the serogroups *Canicola* (7) and *Hardjo Prajitno* (5).

**Figure-3:**
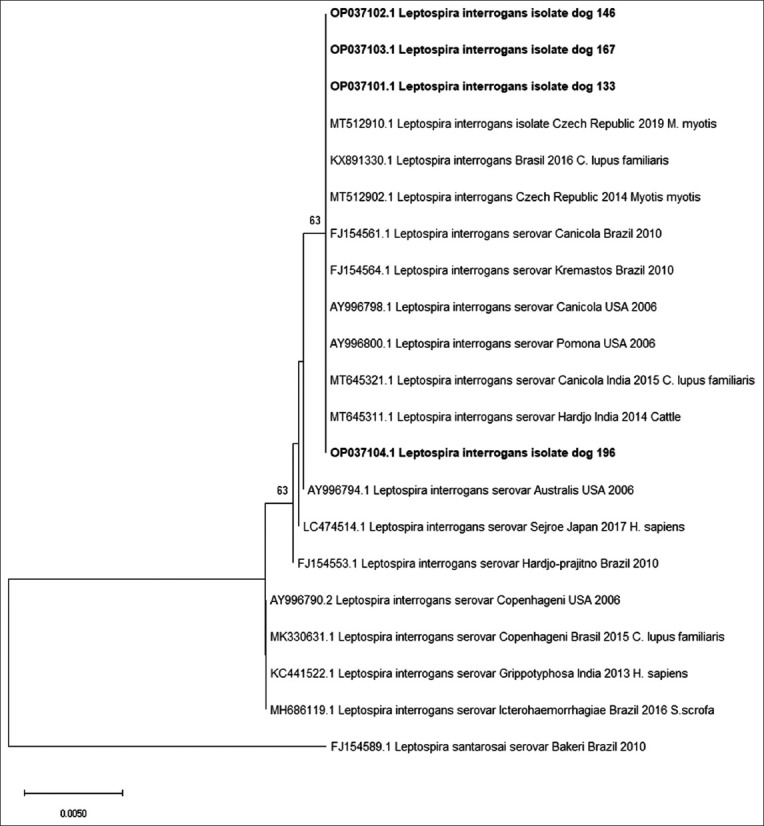
Phylogenetic tree for *Leptospira interrogans* based on 16S rRNA, using the maximum likelihood algorithm and Bootstrap of 1000 replicates, in which values >50 are shown. Taxonomic units are referenced with *L. interrogans* serovars, country, year, and species in which it was detected. The sequences reported in this study are shown in bold and it is evident that they are part of a single cluster with a Bootstrap of 63 where there are also samples from bats (*Myotis myotis*), dog and bovines, from American, European, and Asians.

To construct consensus sequences (133, 146, 167, and 196), four samples with a phred score >20 were obtained, which were aligned with 17 *Leptospira interrogans* serovar sequences using Clustal W (Bioedit). We used MEGA X software to demonstrate that the Kimura 2-parameter model with a neutral support rate was the most applicable evolutionary model. The phylogenetic tree was generated using the ML algorithm and a bootstrap of 1000 replicates. Sequences produced in this study have been submitted to GenBank with accession numbers OP037101, OP037102, OP037103, and OP037104 (Figure-3).

## Discussion

This study showed for the 1^st^ time the infection caused by *Leptospira* spp. in stray dogs and cats, using complementary diagnostic techniques in Colombia to identify previously exposed and actively infectious pets. The molecular diagnosis showed that 56% of the animals were positive for *Leptospira* spp., suggesting that these pets could be infectious, participate in the transmission cycle to humans, and shed the bacteria in their urine or faces. In this sense, PCR as a direct technique was used to diagnose animals in the leptospiremia stage with an important bacteremia load. In the acute phase, this stage occurs in the first 7 days [15]. However, the MAT test showed that 77% of the samples were positive, suggesting that this tendency could be extrapolated to all PCR-positive samples. In this respect, 77% of the animals infected must have been previously exposed to antibodies. Animals that were positive in PCR and MAT may belong to animals who have experienced a new infection and present antibodies from a previous infection with another serovar, which may have a re-infection. Antibodies are generated in the 2^nd^ week and may last between 4 and 6 months or more [15]. Identification of antibody titers >100 for several serovars by MAT can demonstrate multiple exposures to the disease. Animals such as dogs and cats may be susceptible to different serovars; re-infection by other serovars may increase disease transmission; therefore, stray dogs and cat animals can be key reservoirs of the disease in urban areas. Re-infections are associated with environmental, demographic, and individual exposures. It should be noted that the city has favorable climatic conditions, with periods of heavy rains, wetlands, floods, and the presence of rodents, which can further expose roaming animals to the disease [3]. However, further studies are needed to characterize the immune response after infection in dogs and cats, as well as the clinical presentation of the disease during re-infection. In a prospective study conducted in Brazil, re-infection is a very frequent and rapid decline in the humoral response and short-lived immunity that can disappear after 90 days [21].

On the other hand, 23% of the PCR-positive samples were MAT negative, possibly corresponding to animals in the early stages of leptospiremia, where diagnosis by PCR would allow early knowledge of leptospiremia. PCR is a useful tool in acute cases of leptospirosis because it can detect positive cases that other serological tests did not detect [22].

The presence of *Leptospira* spp. in stray dogs has been widely reported; a meta-analysis study on the prevalence of leptospirosis in stray dogs between 1973 and 2019 showed that leptospirosis in stray dogs is present worldwide, with an average prevalence of 27.6%; likewise, a high number of reports in Latin America [23]. Despite the lack of studies about *Leptospira* spp. in stray dogs in Colombia, studies of *Leptospira* spp. in dogs in urban areas of small municipalities reported a prevalence of 21.4%. In the rural area of Córdoba, the prevalence of *Leptospira* spp. in canines from indigenous communities was 47.1% and 79.9%, respectively [24, 25], evidenced that this disease circulates in pets. In Bogota, a prevalence of 36.4% in dogs treated at a veterinary clinic of the Universidad Nacional de Colombia using serological techniques was reported [26]. The presence of *Leptospira* spp. in stray animals in this study was highly significant because they are an important reservoir of leptospirosis, which is a public health problem. In dogs, there were a greater number of positive cases in females, coinciding with the results in Canada [27], which may be associated with street habits such as sniffing and licking the genitals during the heat period of the female. The prevalence of the disease according to age has been discussed; however, differences among ages have not been identified [8]. However, no predominant trend was observed in this study. A review of 476 articles on leptospirosis in stray dogs showed that the most common serovars were *Canicola*, *Icterohaemorrhagiae*, *Grippotyphosa*, and *Pomona* [23], coinciding with the results of the present study, in which *Hardjo Prajitno* was also included, indicating the diversity of serovars present in stray dogs.

In recent years, there has been an increasing concern about the role of cats in the transmission of leptospirosis. To the best of our knowledge, this is the first report of the presence of *Leptospira* spp. in cats in Colombia. The results showed that *Leptospira* spp. was more abundant in cats than in itinerant dogs in Bogota, with higher concentrations in female cats and at early ages; however, few animals were studied due to feral behavior that made it difficult to apprehend them. No cats with high titers were observed, and no acute cases were identified on the basis of MAT. The stage of leptospiremia in cats is short because they quickly generate antibodies; likewise, cats can be asymptomatic [28]. The presence of cats may be related to their behavior and the consumption of rodents; in addition, there is no vaccination for these species. These results show that cats present bacteria, can be exposed to several serovars without acute serological symptoms, and become potential carriers of the disease. Other studies have also reported the presence of *Leptospira* spp. in cats. In Chile, a 15% positivity rate was reported in cats diagnosed by molecular techniques [7]; in Canada, there was a 20% presence in wild cats of Eduardo Principe Island [29]; and in Thailand, a prevalence of 5.4% was reported [30]. *Canicola* (7) and *Hardjo Prajitno* (5) serovars are specific to the dog and bovine species, respectively; however, the presence of different serovars in cats has also been reported in Canada [29].

The diagnosis of the disease is essential to take the most appropriate preventive measures. However, the diagnosis of leptospirosis is complex due to the difficulties of its incubation; the serological diagnosis is effective after the acute phase, and the detection by PCR presents certain challenges [11]. Diagnosis using MAT presents advantages because it allows the identification of the serovar, which has an important epidemiological value, which is one of the limitations of PCR [31]. MAT requires the maintenance of live *Leptospira* spp. cells that represent all serogroups; it may present cross-reactions, the lack of specific antibodies in the immune system or lower Ab titers that can lead to false negative results, and it is also a technique whose results are obtained after several days. A study compared real-time PCR and culture techniques for the detection of *Leptospira* spp. in canines and found that PCR had greater sensitivity and specificity compared to culture; however, as the number of days post-infection increased, both techniques decreased their effectiveness in determining percentage positivity even after day 7 [32]. Culturing *Leptospira* spp. represents a challenge unless it is found at concentrations as high as 10^6^ colonies/mL of sample; however, its slow growth and the conditions that must be maintained for successful replication must be considered [33]. In addition, if the pathogen in the culture is dead, PCR can still detect the presence of DNA. Therefore, complementary diagnoses are necessary to study the epidemiology of leptospirosis.

Sequencing and phylogenetic analysis showed that all the samples were classified as *L. interrogans*. In addition, three of the four samples are grouped in the upper part of the clade closer to the *L. interrogans* reported in *Myotis myotis*; however, the epidemiology of the disease in bats from Bogota is unknown. The other sample analyzed is in the lower part, close to the *Hardjo* serovar reported in India, which suggests a transmission caused by bovines. Therefore, a larger number of samples are suggested to build a tree with results that better reflect reality and phylogenetic relationships.

## Conclusion

To the best of our knowledge, this is the first report of *Leptospira* spp. using complementary techniques in stray dogs and cats in Bogota, DC. Sixty-six percent positivity was determined in animals, which was higher in cats than in dogs. Seventy-seven percent of the samples tested were positive according to MAT. Titers >400 were observed in dogs but not in cats. *Canicola*, *Icterohaemorrhagiae*, *Pomona*, *Hardjo Prajitno*, and *Canicola* and *Hardjo* *Prajitno* serogroups were associated with dogs and cats, respectively. Phylogenetic analysis revealed that the strains belonged to *L. interrogans* serovars related to isolated samples of American, European, and Asian bats (*M. myotis*), dogs, and bovines of American origin.

## Authors’ Contributions

BLGB and NFSC: conceptualization, methodology, validation, formal analysis, resources, data curation, writing – review and editing, software, supervision, and project administration. MMMP and KDJC: conceptualization, methodology, validation, formal analysis, data, curation, YAMR: Methodology, formal analysis, data. GATG: conceptualization, writing – original draft preparation, writing – review and editing, visualization. All authors have read, reviewed, and agreed to the published version of the manuscript.
